# Targeting Nanomaterials to Head and Neck Cancer Cells Using a Fragment of the Shiga Toxin as a Potent Natural Ligand

**DOI:** 10.3390/cancers13194920

**Published:** 2021-09-30

**Authors:** Elena Navarro-Palomares, Lorena García-Hevia, Esperanza Padín-González, Manuel Bañobre-López, Juan C. Villegas, Rafael Valiente, Mónica L. Fanarraga

**Affiliations:** 1 The Nanomedicine Group, Institute Vadecilla-IDIVAL, Molecular Biology and Anatomy & Cell Biology Departments, Faculty of Medicine, University of Cantabria, Avda. Cardenal Herrera Oria, 39011 Santander, Spain; elena-maria.navarro@alumnos.unican.es (E.N.-P.); epadingonzalez@rcsi.ie (E.P.-G.); juan.villegas@unican.es (J.C.V.); 2 Advanced (Magnetic) Theranostic Nanostructures Lab, The Nanomedicine Group, International Iberian Nanotechnology Laboratory, Avda. Mestre José Veiga, 4715-330 Braga, Portugal; manuel.banobre@inl.int; 3 The Nanomedicine Group, Institute Valdecilla-IDIVAL, Applied Physics Department, Faculty of Sciences, University of Cantabria, Avda. de Los Castros 48, 39005 Santander, Spain; rafael.valiente@unican.es

**Keywords:** oral cancer, nanotechnology, toxin, recombinant ligand-protein

## Abstract

**Simple Summary:**

To mimic natural ligand-specific cell entry mechanisms, we have reproduced the molecular cues found in the Shiga toxin to target nanomaterials into head and neck cancer (HNC) cells bearing the globotriaosylceramide receptor (known as GB3 or CD77). This is a globoside typically found in immature endothelial cells of the intestine, also present on the surfaces of many cancerous/precancerous cells of the digestive tract such as HNC. To achieve functional biomimicry, we have coated nanoparticles with a recombinant chimera protein containing the innocuous B domain of the Shiga toxin genetically fused to a nanomaterial-binding sequence. Our results demonstrate that nanomaterials functionalized with this ligand recognize GB3+ve cells reproducing the cellular responses activated by the full-length toxin to sneak into the cellular cytoplasm. These results show how custom biofunctionalization methods can transform inert nanomaterials into hybrid nanosystems capable of identifying specific receptors predictably triggering specific cellular responses as dictated by the proteins in their coating.

**Abstract:**

Head and Neck Cancer (HNC) is the seventh most common cancer worldwide with a 5-year survival from diagnosis of 50%. Currently, HNC is diagnosed by a physical examination followed by an histological biopsy, with surgery being the primary treatment. Here, we propose the use of targeted nanotechnology in support of existing diagnostic and therapeutic tools to prevent recurrences of tumors with poorly defined or surgically inaccessible margins. We have designed an innocuous ligand-protein, based on the receptor-binding domain of the Shiga toxin (ShTxB), that specifically drives nanoparticles to HNC cells bearing the globotriaosylceramide receptor on their surfaces. Microscopy images show how, upon binding to the receptor, the ShTxB-coated nanoparticles cause the clustering of the globotriaosylceramide receptors, the protrusion of filopodia, and rippling of the membrane, ultimately allowing the penetration of the ShTxB nanoparticles directly into the cell cytoplasm, thus triggering a biomimetic cellular response indistinguishable from that triggered by the full-length Shiga toxin. This functionalization strategy is a clear example of how some toxin fragments can be used as natural biosensors for the detection of some localized cancers and to target nanomedicines to HNC lesions.

## 1. Introduction

Head and neck cancer (HNC), often referred to as squamous cell carcinomas of the head and neck, is the seventh most common cancer in the world, representing approximately 3% of all cancers [1,2]. Typically associated with the heavy use of tobacco and alcohol in older patients, and with human papillomavirus among younger people, HNC includes a marked heterogeneity of tumors arising from the mucosal surfaces of four major anatomical sites: the oral cavity, sinonasal cavity, pharynx, and larynx [3]. 

The treatment indicated for accessible tumor masses is surgical, often followed by adjuvant radiotherapy or chemoradiotherapy [1]. However, unfortunately, local recurrence of HNC squamous cell carcinoma is common [4,5]. The poor physical detection of the margin of the cancerous lesion, surgically inaccessible tumors, or second primary tumors often determine the prognosis of HNC. As a result, only 40–50% of HNC patients survive 5 years after diagnosis [4,5]. New technologies, such as targeted nanomedicine, could help to improve both cell detection and selective cell killing.

**Figure 1:**
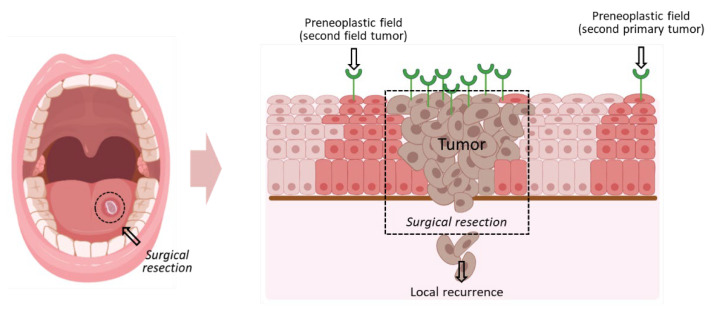
HNC lesion cell and GB3 receptor distribution in precancerous and malignant HNC cells. Diagram of the relationship between clinically identifiable HNC lesion (circled) and clinically invisible precursor fields of cancerous tissue (in red). The surrounding preneoplastic cells are the source of local recurrences (which develop from the primary tumor) and second primary tumors (from a second field) after surgical resection of the initial carcinoma. These areas are indicated in the diagram. The expected expression of the GB3 receptor (in green) in these regions is shown. Created with BioRender.com.

One of the obstacles in the early diagnosis of oral cancer is the lack of specific early markers to unequivocally identify the cancerous/pre-cancerous lesions, and more particularly, the surgical margins during the intervention (Figure 1) [6]. These facts render the treatment ineffective, resulting in a high recurrence rate. Therefore, a key issue in HNC pathogenesis is the identification of the preneoplastic fields of the mucosal epithelium. These contain genetically altered cells, related to carcinoma, that often extend into the surgical margins when tumors are excised, often causing local recurrences and secondary primary tumors [7,8].

The discovery of the role of epidermal growth factor receptor (EGFR) overexpression in the pathogenesis of HNC has opened new pathways in the development of novel therapeutic agents for the targeted treatment of recurrent and/or metastatic head and neck squamous cell carcinoma. Cetuximab is a monoclonal antibody inhibitor of the EGFR that has produced impressive therapeutic benefits that account for a significantly prolonged locoregional control and medial survival, compared with radiotherapy alone. The addition of cetuximab to radiotherapy reduces by 32% the risk of locoregional failure and a 26% risk of death resulting in a nearly 20 month increase in median survival with the addition of cetuximab to radiotherapy [9]. However, acquired and innate resistances have precluded current anti-EGFR agents from offering sustainable benefits to initially responsive cancers and benefits to EGFR-positive cancers that are inherently resistant [10].

Thus, the concept of immunotherapy has shifted from immune modulation to tumor-antigen-specific therapy realizing a targeted oncologic therapy taking advantage of newly discovered tumor-associated antigens. However, in patients with HNC, many immunosuppressive mechanisms are activated and relevant effector cells are suppressed in their function, hampering any form of immunotherapy [11].

In the search for new ways to selectively identify and treat HNC, we seek specific ligands on the surface of these cells to target diagnostic and/or therapeutic nanoparticles. Glycans are among the possible markers since they play roles in tumor cell signaling, dissociation and invasion, cell-matrix interactions, angiogenesis, metastasis, and immune modulation [12,13,14,15]. Most cancer cells, including HNC cells, show altered surface membrane protein glycosylation patterns. Furthermore, aberrations in glycosylation patterns are part of the “hallmark of cancer” [14].

The glycosphingolipid globotriaosylceramide (GB3 or CD77) is a globoside typically found in the immature endothelial cells of the intestine (Figure S1) [16,17] that has also been detected on the surfaces of many cancerous/precancerous cells of the digestive tract, including HNC [18,19]. This carbohydrate is exceptionally interesting because it is the receptor of the Shiga toxin (ShTx), a potent bacterial natural ligand that has been used to target and destroy GB3-positive cancer cells [19,20,21,22,23,24,25,26,27,28] and to boost local anticancer immunity [23]. Interestingly, ShTx binding to the GB3 receptor penetrates the cell retrogradely, circumventing the endo-lysosomal vesicular pathway, thus, preventing the degradation of the toxin [24,29,30,31,32,33]. This “non-canonical” receptor-mediated entry route could represent a new interesting way to introduce nanosystems directly to the cell’s cytoplasm, preserving the properties of different nanosystems. Here, we propose to use biotechnology to target nanoparticles locally to HNC tissues by replicating the molecular signals used by ShTx.

## 2. Materials and Methods

### 2.1. Nanoparticle Synthesis, SiO_2_ Coating and Characterization

Monodispersed SiO_2_ spheres (~500 nm, Figure S2) were prepared using a modified Stöber method as previously described [34]. A tetraethyl orthosilicate (TEOS) solution was added to a solution containing ammonium hydroxide, water, and ethanol, which was stirred at room temperature for 2 h. The excess of reagents was removed by 3 centrifugation/redispersion cycles in ethanol (3300 g, 10 min). The resulting particles had a ζ-potential of −28.1 ± 0.5mV. For in vivo imaging purposes we used carboxylate-modified red fluorescent polystyrene particles (~500 nm) purchased from Sigma-Aldrich (ζ-potential −44.0 ± 0.3 mV).

For in cellulo studies and electron microscopy visualization of the nanoparticles samples we synthetized Fe_3_O_4_@SiO_2_ nanoparticles with Rhodamine B isothiocyanate (RBITC). Hydrophobic iron oxide NPs were prepared by a hydrothermal approach as previously described [35]. For the fluorescent-SiO_2_ coating, an adapted reverse microemulsion method was described previously [36]. The reverse microemulsion system was prepared by mixing Igepal CO-520, cyclohexane, the ferrofluid, ammonium hydroxide solution (NH_4_OH) (all from Sigma-Aldrich), the RBITC-APMS conjugate, and TEOS that were sequentially added. Ethanol was added to disrupt the microemulsion and precipitate the NPs. The resulting Fe_3_O_4_@SiO_2_:RBITC particles had a ζ-potential of −52.1 ± 1.5 mV. All nanomaterials were ethanol-dispersed, deposited onto a Lacey copper grid, and characterized using a JEM 1011 (JEOL) transmission electron microscope (Figure S2).

### 2.2. Gene Synthesis, Protein Expression and Purification

The designed ShTxB:6xHis recombinant gene constructs were synthesized by General Biosystems, Inc. (Morrisville, NY, USA) and were cloned in pET-15b plasmid systems (Novagen, Merck KGaA, Spain). One Shot™ BL21(DE3) *E. coli* (NZYTech, Portugal) cells were transformed with the expression vectors. Bacterial cultures were grown in Luria-Bertani (LB) broth supplemented with 100 μg/mL of ampicillin and 35 μg/mL of chloramphenicol until A_600_ ca. 0.6. The expression of the protein was induced by adding 0.1 mM of isopropyl b-D thiogalactopyranoside (IPTG, PanReac, AppliChem). Cells were collected after 4 h by centrifugation and were resuspended in 50 mM NaPi, 300 mM NaCl, pH 8.0 with 1 mg/mL lysozyme, and protease inhibitors (Pierce, Thermo Fischer, Spain).

Bacterial cell lysates were obtained by probe sonication (5 × 15 s pulses at 130 W, 65% amplitude, with 15 s intervals, at 4 °C) and insoluble material was removed by centrifugation. Bacterial soluble protein lysate was loaded onto pre-equilibrated Ni-TED columns (Protino^®^ Ni-TED, Macherey-Nagel GmbH & Co., Düren, Germany). The recombinant His-tagged protein was eluted in a buffer supplemented with 250 mM imidazole. Finally, PD-10 desalting Columns (GE Healthcare, Chicago, IL, USA) were used to remove the imidazole and to exchange the buffer to PBS.

### 2.3. FITC Protein Labeling

ShTxB:6xHis and serum proteins were labeled with fluorescein isothiocyanate (FITC, Sigma-Aldrich). The protein solution in PBS was first treated with 1M sodium bicarbonate buffer, pH 8.8, adding 0.1 mL per 1 mL of protein. Then 50 μL of 5 mg/mL FITC solution in DMSO were slowly added under continuous stirring, and the reaction was kept for 1 h at room temperature. The labeled protein was separated from the unconjugated FITC using gel filtration (Sephadex^®^ G-25 resin) PD-10 columns (GE Healthcare, Chicago, IL, USA).

### 2.4. Protein Bioconjugation on Particles, SDS-PAGE Protein Analysis

The bioconjugation experiment has been fully described in previous works [37]. The technique consists of 3 tip sonication cycles of 2 s of the particles in phosphate-buffered saline (PBS) containing excess amounts (ca. 0.5 mg/mL) of ShTxB protein (Figure S3). Functionalized nanomaterials were washed 3 times with PBS, through centrifugation/redispersion cycles, to remove the excess protein. For SDS-PAGE, the protein functionalized on the particles was stripped using a Laemmli sample buffer (BioRad) at 95 °C for 2 min. SDS-PAGE electrophoresis was performed using Mini-Protean^®^ precast gels (BioRad). Protein analysis was performed on Coomassie blue-stained gels that were scanned using the BioRad GelDoc EZ system.

### 2.5. Cell Culture and Staining, Fluorescent Confocal and Electron Microscopy Imaging

Detroit 562 cells, human pharynx epithelial carcinoma cells derived from a metastatic pleural effusion, were obtained from ATCC Ref CCL-138. Cultures were grown in Eagle’s Minimum Essential Medium (Biowhittaker™) containing 10% bovine serum. HaCaT cells, human epidermal keratinocyte line, were obtained from Drs. Jorcano and Quintanilla’s laboratory and were grown in Iscove’s Modified Dulbecco’s Medium containing 10% bovine serum. Both cell lines were kept in standard conditions. Cells were exposed to approximately 5 µg/mL of bioconjugated particles for 24 h. Cells were fixed with 4% paraformaldehyde, and were stained with Hoechst 33258 (Sigma-Aldrich^®^, San Luis, CA, USA), Acridine Orange (Sigma-Aldrich^®^), or LysoTracker Deep Red (Thermo Fisher, Waltham, MA, USA). Alexa647-anti-Human CD77 (BD Pharmingen™, San Diego, CA, USA) was used for immunostaining the GB3 receptor. Confocal images were taken with a Nikon A1R microscope. All fluorescent images are pseudo-colored. Cell samples processed for electron microscopy were fixed with 3% glutaraldehyde in 0.12 M PBS for 24 h and were post-fixed in 2% buffered osmium tetroxide, dehydrated in a graded acetone series, and embedded in Araldite. Ultrathin sections of ca. 70 nm thick, were obtained on an LKB ultramicrotome, stained with lead citrate and uranyl acetate. TEM was performed using a JEOL JEM 1011 operated at 100 kV. Diagrams have been drawn using the BioRender software tool.

### 2.6. Preclinical Murine Models

In vivo experiments were designed and performed to minimize the use of animals. C57BL/6 mice (12 weeks old) were housed with a 12 h light/dark cycle with free provision of food and water at the Experimentation Service (SEEA) of the University of Cantabria. The animals were maintained, handled, and sacrificed following the directive 2010/63/UE. The preclinical model of oral carcinogenesis was produced using the carcinogen 4-Nitroquinoline-1-oxide (4NQO, Sigma Aldrich) as previously described [38,39,40]. In brief, C57Bl/6 mice were drinking water with 100 µg/mL of 4-NQO for 16 weeks (water was changed once a week). After 16 weeks of carcinogen treatment, the mice were analyzed for precancerous and cancerous lesions in the oral cavity. Then, the mice were supplied with water containing 5 µg/mL of the PS@ShTxB particles for 10 min. The mice were humanely euthanized, the tongues were dissected and photographed. Fixed tissues were embedded in sucrose and frozen in an optimal cutting temperature compound (OCT, Tissue Tek) for 6 μm with cryostat sectioning.

## 3. Results

### 3.1. Innocuous Shiga-Toxin Ligand Design and Production

**Figure 2:**
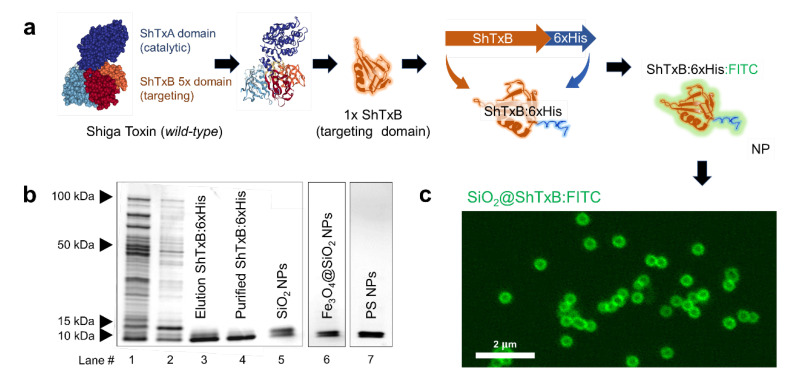
Ligand protein design. (**a**) Different domains in the structure of the Shiga toxin (ShTx). A single B domain (ShTxB) colored in orange is shown (UniProtKB ref. P09386). A diagram of the genetically modified ShTxB chimera protein and its situation on the nanoparticle is shown. The electrostatic interaction of the 6xHis tail orients the ligand peripherally on the particle. (**b**) SDS-PAGE analysis of ShTxB protein at different production and purification steps. (Lane# 1) protein expression in bacteria, (#2) column wash, (#3) elution of the protein from the column with imidazole, and (#4) PD-10 purified ShTxB used for bioconjugation. Lanes #5-6 show ShTxB stripped from functionalized SiO2, Fe_3_O_4_@SiO_2_, and PS particles (#7). (**c**) Confocal microscopy single Z plane image of 500 nm SiO_2_ particles bioconjugated with ShTxB coupled to FITC. The fluorescent halo around the particle is the FITC-stained recombinant protein.

The ShTx is composed of two different polypeptides, the catalytic “A” domain, and the targeting ligand, the “B” domain. The active toxin is constituted when five “B” subunits recognize and bind the GB3 receptor on the target cell membrane, and assemble a homopentamer where a single polypeptide “A” couples (Figure 2a). This provokes membrane ruffling enabling toxin cell entry [29,30,31,32,33,41]. Hence, the “B” subunit of the toxin by itself is a potent natural ligand that is innocuous as a monomer [25,27,28,42].

Here we use biotechnology to produce a new synthetic protein containing a single “B” subunit of the Shiga toxin (that we named ShTxB), genetically attached to a cationic peptide (6xHis-tag) to electrostatically functionalized nanomaterials following a method we have previously described and characterized (Figure S3) [37]. This peptidic cationic tail creates an electrostatic zipper with the negative surface of the particles that allows oriented positioning of the ShTxB ligand on the nanoparticle surface (Figure 2a). Moreover, the binding method is highly stable upon exposure to physiological conditions (for times longer than 72 h) and significantly hinders non-specific protein biofouling [37].

The ShTxB ligand-protein was produced in bacteria and was purified as described in the Materials section (Figure S4). Upon functionalization, the binding of the ShTxB to different nanoparticles was monitored using SDS-PAGE analysis (Figure 2b). As a proof-of-concept, silica particles of ca. 500 nm and ζ-potential of ca. −20 mV were coated with the synthetic ShTxB protein and imaged using confocal microscopy. Figure 2c shows ShTxB conjugated with fluorescein isothiocyanate (FITC) as a fluorescent halo on the surface of the particles.

### 3.2. Shiga-Toxin Receptor GB3 Is Overexpressed in Head and Neck Cancer Cells

To carry out the in vitro (in cellulo) experiments we chose two human epidermal cell lines. As a control, we chose the immortalized human keratinocyte cell line HaCaT that is often used as a standard model of epidermal cells [43]. As a model of human malignant HNC, we used Detroit 562 cells, derived from the pleural fluid of a patient with primary carcinoma of the pharynx [44].

To determine the levels of expression and the distribution of the GB3 receptor on the surface of the cells, we performed immunocytochemistry on live cells. Confocal microscopy cell images demonstrated different levels of GB3 expression on the surfaces of these cell lines. We found that while HaCat cells occasionally exhibited small spots of GB3 on the surface, malignant cells were conspicuously positive (Figure S1B). Quantitative flow cytometry analysis revealed that a small percentage of the HaCat control cells (approximately 16%) exhibited the receptor on their surfaces, compared to 84% on the malignant HNC cells. These results suggest that, although HaCaT cells are normally taken as the model for normal epithelial cells, some of these cells could be in a precancerous stage. This study validates these two cell lines as suitable models for the study of nanoparticle interaction with GB3.3.3. 

### 3.3. ShTxB-Coated Particles Specifically Interact with GB3 Triggering Receptor Clustering 

The affinity of the ShTxB-functionalized particles for the GB3 receptor was analyzed using confocal microscopy imaging. To visualize the particles, we prepared two types of nanomaterials, 500 nm diameter SiO_2_ particles for fluorescent-optical microscopy, and Fe_3_O_4_@SiO_2_ nanoparticles for fluorescent and electron microscopy (see below). Both particles were coated with FITC-labelled ShTxB (SiO_2_@ShTxB:FITC) and were added to HNC cell cultures at a concentration of 5 µg/mL and the attachment of the protein to the surface of the two types of nanoparticles was confirmed using SDS-PAGE analysis (Figure 2b, lane #5). For colocalization purposes, cells were simultaneously live immunostained with antibodies recognizing GB3.

**Figure 3:**
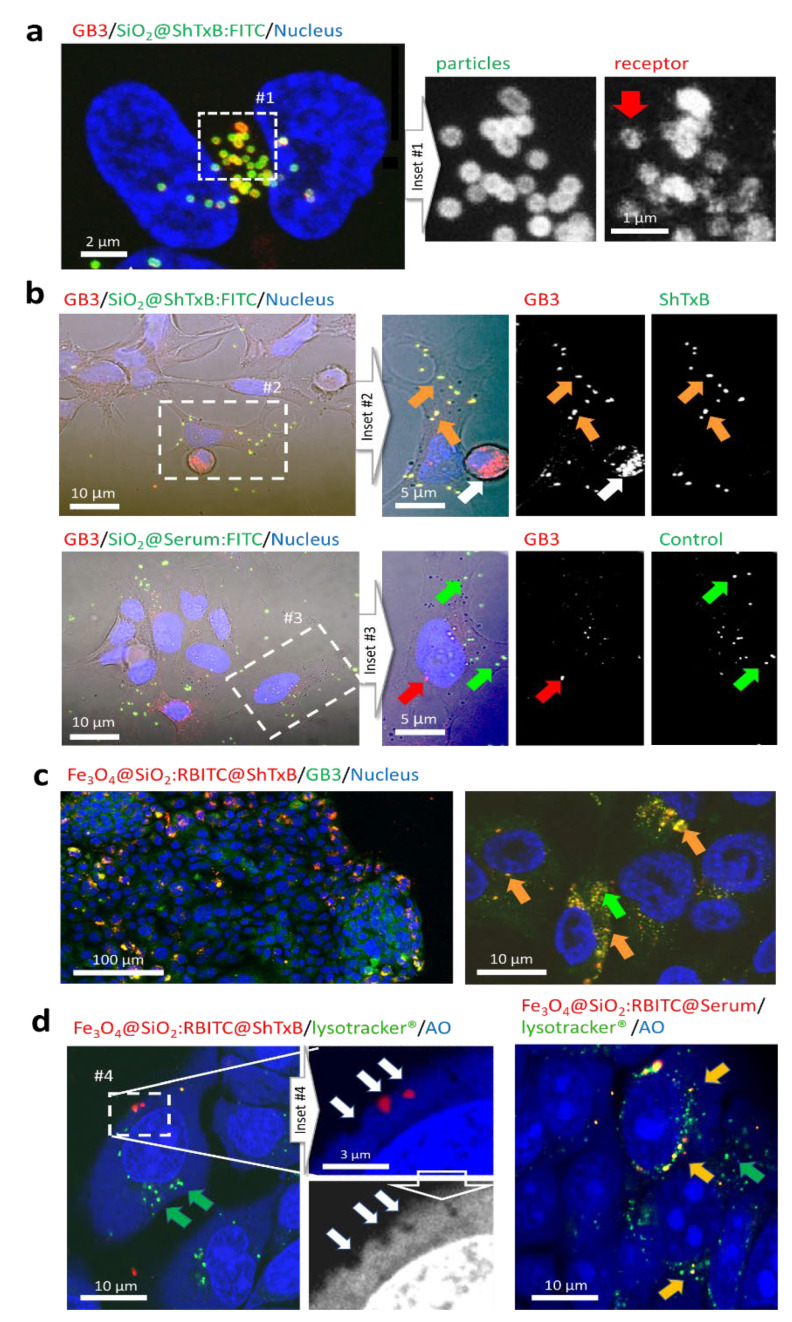
Targeting of ShTxB-coated particles to live HNC cells. (**a**) Live human HNC cells exposed to SiO_2_@ShTxB:FITC particles (green channel). The clustering of GB3 receptors (inset #1, red arrow) is observed. (**b**) Phase-contrast/fluorescent confocal microscopy images of cells exposed to particles functionalized with ShTxB or serum for 1 h. All ShTxB-particles colocalize with GB3 receptors (inset #2, orange arrows). A rounded cell, undergoing cell division (inset #2, white arrow), displays no surface GB3 receptors and serves as a negative control. (inset #3, green arrows) Serum-coated particles are randomly distributed in the extracellular and intracellular spaces. (orange arrows). (**c**) Colocalization of Fe_3_O_4_@SiO_2_:RBITC@ShTxB nanoparticles and GB3 receptors. (**d**) Confocal single Z plane images of ShTxB-coated and control serum-coated nanoparticles on cells stained with acridine orange (AO, blue channel) a lysosomal marker (lysotracked^®^, green channel). Clusters of ShTxB- nanoparticles are trapped in membrane folds during cell entry (red channel, white arrows) and do not colocalize with the lysosomes (labeled in green, green arrows). On the contrary, serum-coated nanoparticles colocalized with lysosomes (orange arrows). Green arrows point at empty lysosomes.

Figure 3 shows high-resolution fluorescent microscopy images of some HNC cells treated with the ShTxB-coated particles (green channel). Confocal microscopy imaging shows how all observed particles colocalize with GB3 receptors (red channel). The SiO_2_@ShTxB:FITC particles appeared to recruit and cluster the GB3 receptors around them (inset#1, red arrow) producing a “receptor clustering effect” similar to that described for the wild-type toxin on target cell membranes. This suggests the biological coating of the particles could behave biomimetically [29,31].

To further check particle targeting, we compared the colocalization of SiO_2_@ShTxB:FITC particles with that of identical particles coated with serum proteins (SiO_2_@Serum:FITC). Confocal microscopy imaging revealed that, while 100% of the SiO_2_@ShTxB:FITC particles localized on GB3 receptors (inset #2, orange arrows), serum-coated particles were distributed at different points on the cell surface where there was no detectable GB3 (inset #3, green/red arrows).

To corroborate these results in a different system, we prepared a nanocomposite that consisted of iron oxide nanoparticle cores coated with amorphous silica shells containing rhodamine isothiocyanate dye (Fe_3_O_4_@SiO_2_:RBITC), a red fluorophore [36]. As in the previous experiment, these nanoparticles were bioconjugated with the purified ShTxB following an identical procedure. The attachment of the protein to the surface of the Fe_3_O_4_@SiO_2_:RBITC@ShTxB nanoparticles was confirmed using SDS-PAGE analysis (Figure 2b, lane #6). These nanoparticles were added to live cultures of HNC cells at a concentration of 5 µg/mL and were incubated for 2 h before cell fixation and image processing.

Clumps of Fe_3_O_4_@SiO_2_:RBITC@ShTxB nanoparticles can be readily detected in cells by confocal and electron microscopy techniques [36]. Confocal microscopy examination of the cells confirmed that the nanoparticles also co-localized with GB3 receptors (Figure 3c, orange arrows), thus supporting previous results and demonstrating the affinity of the recombinant ShTxB ligand for the GB3 receptor. As for the SiO_2_ particles, these nanocomposites also crossed the membranes in lysosome-independent membranous compartments (white arrows). Hence, these results show that the ShTx “B” domain is a potent natural ligand with a high affinity for GB3 that can be used to identify cancerous and precancerous human HNC cells and to target nanomaterials to these cells.

### 3.4. ShTxB-Coated Nanoparticles Invade Cells Avoiding the Endo-Lysosomal ‘Canonical’ Route

The GB3 receptor non-canonical cell-entry route allows the wild-type ShTx to retrotranslocate to the cell cytoplasm circumventing the receptor-mediated endo-lysosomal vesicular pathway. To sneak into the cell, ShTx causes high cytoplasmic membrane activity using the folds and undulations of the membrane as a “back door” [29,30,31]. Thus, to investigate if the ShTxB-coated nanoparticles trigger a similar cellular response, we used live-cell imaging to identify the endo-lysosomal compartment and the intracellular location of the nanoparticles 3 h after exposure. Confocal microscopy imaging (Figure 3d) confirmed that Fe_3_O_4_@SiO_2_:RBITC @ShTxB nanoparticles (red channel) did not co-localize with lysosomes stained with Lysotracker^®^ (green arrows), while all control serum-coated nanoparticles were included in these vesicles (orange arrows). This experiment also confirmed the presence of deep grooves on the surface of the cell membrane, indicative of intense membrane activity, where groups of ShTxB-functionalized nanoparticles were localized, thus suggesting a possible biomimetic cell entry mechanism (inset #4, white arrows).

### 3.5. Biomimetic ShTxB-Coated Nanoparticle Retrotranslocation

**Figure 4:**
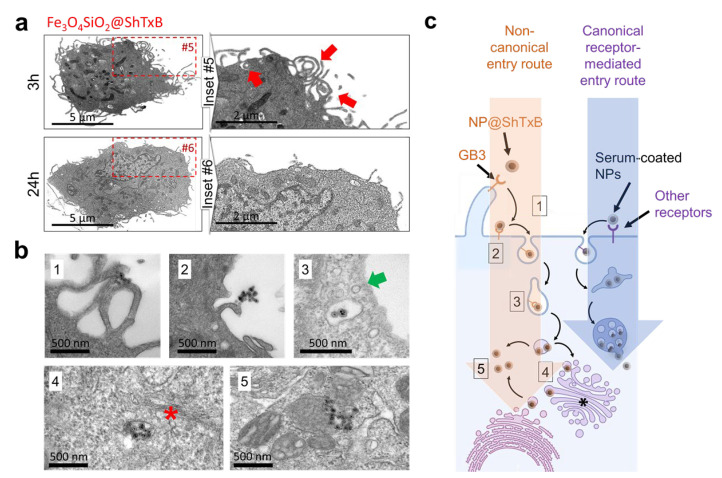
Biomimetic retrotranslocation of the ShTxB-coated NPs. (**a**) TEM images of sections of human HNC cells exposed to ShTxB-coated nanoparticles for 3 h (top) or 24 h (bottom). (inset #5) Functionalized nanomaterials unchain a huge membrane activity upon cell contact (red arrows). This membrane activity is transitory, being restored upon particle cytoplasmic invasion (inset #6). A control cell can be seen in Figure S5. (**b**) Localization of the ShTxB-coated nanoparticles by TEM. The following numbers correspond to those in the diagram in (**c**). (#1) The nanomaterials are being contacted by filopodia. (#2) Filopodia retraction and nanoparticle transport towards the cell surface. (#3) Nanoparticle engulfment in large irregular membranous structures different from conventional endocytic vesicles (green arrow). (#4) Nanoparticle association to membranes resembling the endoplasmic reticulum and Golgi apparatus (asterisk). (#5) Nanoparticles in the cytosol devoid of membranes, intermingling with the cellular organelles. (**c**) Diagram of the proposed non-canonical cell entry route observed for ShTxB-coated nanomaterials. The ‘non-canonical’ route is triggered by the interaction of the ShTxB-coated nanoparticles with the GB3 receptors. This unchains exacerbated membrane activity, leading to nanomaterial retrotranslocation via endoplasmic reticulum to Golgi apparatus (asterisk). Boxed numbers correspond to the stages indicated in Figure 4c.

To further explore the intracellular fate of the bio-coated nanomaterials, we used TEM imaging on ultrathin sections of cells incubated with ShTxB-functionalized Fe_3_O_4_@SiO_2_ nanoparticles. The electron-dense core of the particles is particularly well suited for this purpose. The initial exposure of the HNC cells to the ShTxB-coated nanoparticles unchained an intense membrane activity (Figure 4a). Typically, cells exposed to ShTxB-coated nanoparticles display abundant actin-rich plasma-membrane processes, such as filopodia, lamellipodia, and membrane ruffles (inset #5, red arrows) closely reproducing the membrane response described for the wild-type toxin [30,31,33]. Interestingly, 24 h after this initial contact the membrane returned to normal shape (inset #6).

Based on previous studies [29,30,31], TEM imaging suggested the following sequential steps in the intracellular trafficking of the Fe_3_O_4_@SiO_2_@ShTxB nanoparticles (Figure 4b). Initially, direct contact of the nanoparticles with the membrane surface is observed (step #1). Upon contact, the filopodia of the cell appeared to actively interact with the nanoparticles (step #2). Interestingly, the nanoparticles appeared to be captured and dragged towards the cell body by filopodia as it occurs for natural ligands (such as viruses) (step #2).

In the cytoplasm, ShTxB-hybrid nanoparticles were observed inside membranous compartments with irregular shapes close to the cell cortex. These compartments displayed no underlying visible coatomer covering (i.e., clathrin or similar) compared to typical endocytic vesicles (step #3, green arrow), suggesting ShTxB nanoparticles might use recycling membranes to invade the cytoplasm, as described for the full-length toxin [24,29,30,31]. In some images the internalized nanoparticles also presented a close interaction with the smooth endoplasmic reticulum and the Golgi apparatus (step #4, asterisk), thus suggesting nanoparticle retrotranslocation into the cytoplasm was occurring via these compartments. Some nanoparticles also appeared in the cytosol, intermingling with the cellular organelles and devoid of membranes (step #4). Altogether, these TEM images taken at fixed time points suggest ShTxB-coated nanoparticles did not follow the endo-lysosomal canonical receptor-mediated endocytosis. Findings summarized in Figure 4c, suggest these coated nanoparticles specifically reproduce the cellular response prompted by the natural toxin, unchaining a frantic membrane activity that drives nanoparticles into the eukaryotic cytosol mimicking tricks used by some viruses and bacteria [34,45]. Furthermore, this functionalization also appears to prompt the escape of the nanomaterials into the cytosol following the route used by the wild-type toxin, targeting the nanoparticles at both cellular and subcellular levels.

### 3.6. ShTxB-Coated Particles Recognize HNC Lesions and Precancerous (the Leukoplakia) Zones in Animal Models

**Figure 5:**
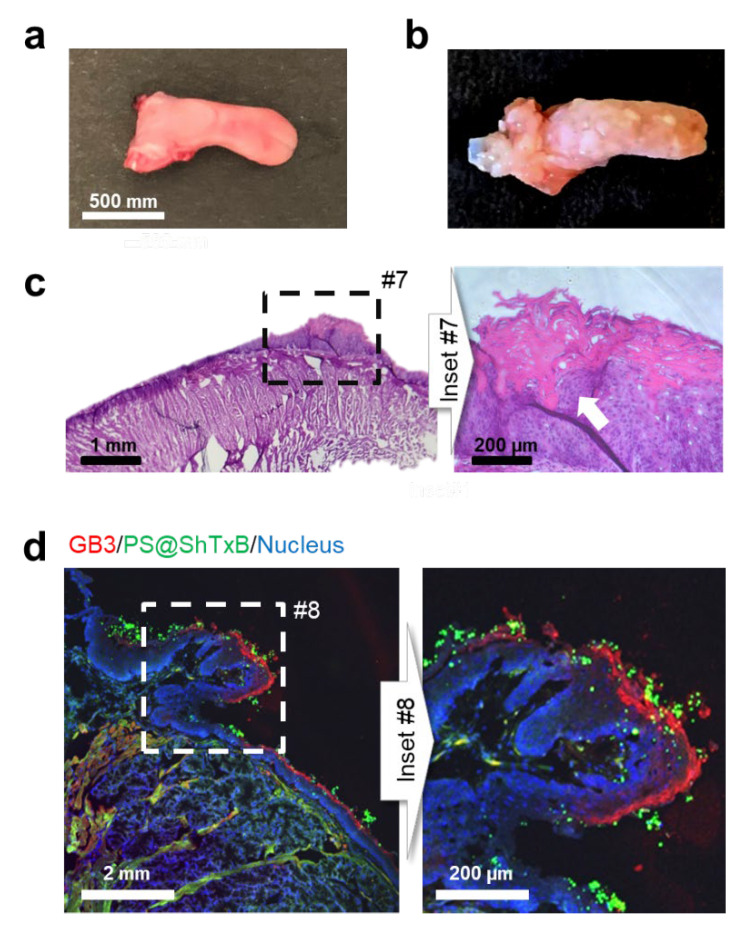
In vivo ShTxB-coated particle targeting. Macroscopic appearance of tongues of control (**a**) and 4NQO-treated mice (**b**). Macroscopic lesions, characteristic of squamous cell carcinoma, are visible on the oral mucosa. Leukoplakia and papilloma-like tumoral masses are distributed throughout the surface of the tongue. (**c**) A hematoxylin-eosin-stained section in a tongue in mice treated with 4NQO where severe dysplasia and an invasive squamous cell carcinoma (inset #7, white arrow) are observed. (**d**) Confocal microscopy image of a cryosection of the affected tissue where a tumoral mass is boxed (inset #8). GB3 receptor immunostaining (red channel) is observed on the surface of the lesions. Upon incubation with the PS@ShTxB (see Figure 2b) particles (green channel) for 10 min, the surface of the GB3-positive lesion is conspicuously coated with the ShTxB-coated nanoparticles. The cell nuclei are visible in the blue channel. These results suggest that this technique can be improved by using different types of nanoparticles to optimize their targeting and thus, be able to selectively apply treatments such as magnetic or optical hyperthermia.

To investigate whether these results will be reproducible in the oral mucosa affected by HNC, we used a well-established and widely-used preclinical mouse model of oral carcinogenesis (see Methods). This model shows a close similarity to human oral carcinogenesis at both histological and molecular levels, and accurately reproduces the histological and molecular changes in the tissues including, hyperplasia, dysplasia, severe dysplasia, and finally, in situ carcinoma (Figure 5) [38,39,40]. As described for the model, histopathological analysis of mice treated with the carcinogen 4NQO (Figure 5b,c) displayed lesions typical of oral squamous cell carcinoma, including leukoplakia, squamous dysplasias, and papillomas in the tongue epithelium, where regions of invasive squamous cell carcinomas were observed (inset #5).

To test the specificity of the ShTxB coating, anesthetized mice were administered red-fluorescent ShTxB-coated particles (PS@ShTxB, see Figure 2b) (at 5 µg/mL) resuspended in water directly in the mouth for 10 min. Upon sacrifice, the tongues of the mice were photographed and processed. Tissue sections were immunostained with the anti-GB3 antibody. Confocal microscopy imaging of the immunostained sections (Figure 5d) demonstrated the GB3 receptor concentrated on the surface of the putative papilloma. As hypothesized, the PS@ShTxB particles (green channel) also clustered around the lesion. In summary, this experiment supports the results obtained in cell lines and the potential use of the ShTxB ligand as an early diagnostic marker to identify cancerous/precancerous lesions and target therapeutic nanoparticles to HNC tissues.

## 4. Discussion

HNC is one of many locally accessible cancers that can benefit significantly from the topical use of nanomedicine, concentrating the therapeutic potential at the site of injury and avoiding the potential side effects of the systemic spread of drugs or nanomaterials. Our results validate GB3 as a receptor of precancerous/neoplastic HNC cells, and ShTxB as a ligand to selectively identify the malignant cells. We also show how this ligand can drive nanoparticles into the cytoplasm of these cells, avoiding the hostile environment of the endo-lysosomal pathway that often destroys nanomedicines. Furthermore, this functionalization also prompts the escape of the nanomaterials into the cytosol, targeting the nanodevices at both cellular and subcellular levels.

The described functionalization strategy can also serve to create precision nanotools for the diagnosis and treatment of at least one-third of the digestive tract neoplasias, where this receptor is abundantly expressed. We hope this new cell recognition, entry, and targeting mechanism could be used shortly in drug delivery or trigger local hyperthermia as an adjuvant to other traditional cancer therapies.

## 5. Conclusions

These findings show how simple molecular cues from nature can be used to transform nanomaterials into bioactive particles capable of binding a specific receptor and triggering unique biomimetic and programmed cellular reactions. The use of both fluorescent and magnetic ShTxB-coated nanoparticles could substantially improve the identification of the margins of HNC lesions. This will improve the early treatment and prognosis of this cancer, preventing local recurrences from the macroscopically normal-appearing epithelium, or lesions with poor anatomical and surgical accessibility.

## Supplementary Materials

The following are available online at https://www.mdpi.com/article/10.3390/cancers13194920/s1, Figure S1: (a) Confocal microscopy image of a mouse intestine cryosection immunostained for the GB3 membrane receptor. (b) Human precancerous and HNC cells immunostained for the GB3 receptor. Figure S2: Transmission Electron Microscopy images of the nanoparticles used, Figure S3: Ligand-protein production and nanomaterial functionalization procedure, Figure S4: Coomassie-blue stained SDS-PAGE electrophoresis gel showing the different fractions of proteins obtained during the overexpression and purification steps of the StxB:6xHis protein (ShTxB). Figure S5: Transmission Electron Microscopy image of an ultrathin section of a control human HNC cell.
