# Supplementary material for: Targeting Nanomaterials to Head and Neck Cancer Cells Using a Fragment of the Shiga Toxin as a Potent Natural Ligand
Source: Cancers (Basel). 2021 Sep 30;13(19):4920. doi: 10.3390/cancers13194920 (PMC8507991; doi:10.3390/cancers13194920)
Supplement: Supplementary file 1 [file cancers-13-04920-s001.zip › cancers-1386916-supplementary.pdf]

Supplementary Materials: Targeting Nanomaterials to Head and Neck Cancer Cells using A Fragment of the Shiga Toxin as A Potent Natural Ligand

Elena Navarro-Palomares [1], Lorena García-Hevia [1,*], Esperanza Padín-González [1], Manuel Bañobre-López [2], Juan C. Villegas [1], Rafael Valiente [1] and Mónica L. Fanarraga [1,*]

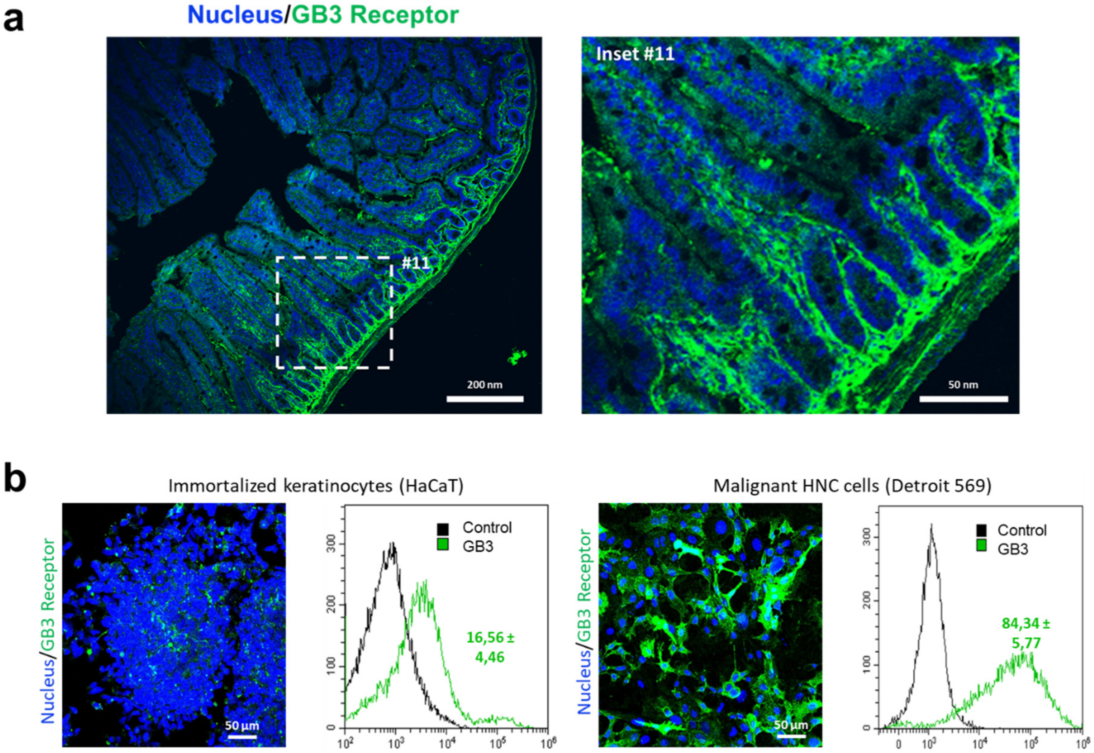

Figure S1. (a) Confocal microscopy image of a mouse intestine cryosection immunostained for the GB3 membrane receptor (green channel). Nuclei are stained with Hoechst (blue channel). Confocal images are pseudo-colored. (b) Human precancerous (Immortalized keratinocytes, HaCat) and HNC cells (Detroit 569) immunostained for the GB3 receptor (green channel). The morphology of the HNC cells in culture shows a mesenchymal shape. Graphs on the right show the quantification of GB3 expression in a total of 10.000 cells by flow cytometry. GB3 positive cells represent ca. 16% in HaCat keratinocytes compared to ca. 84% in HNC Detroit 569 cells.

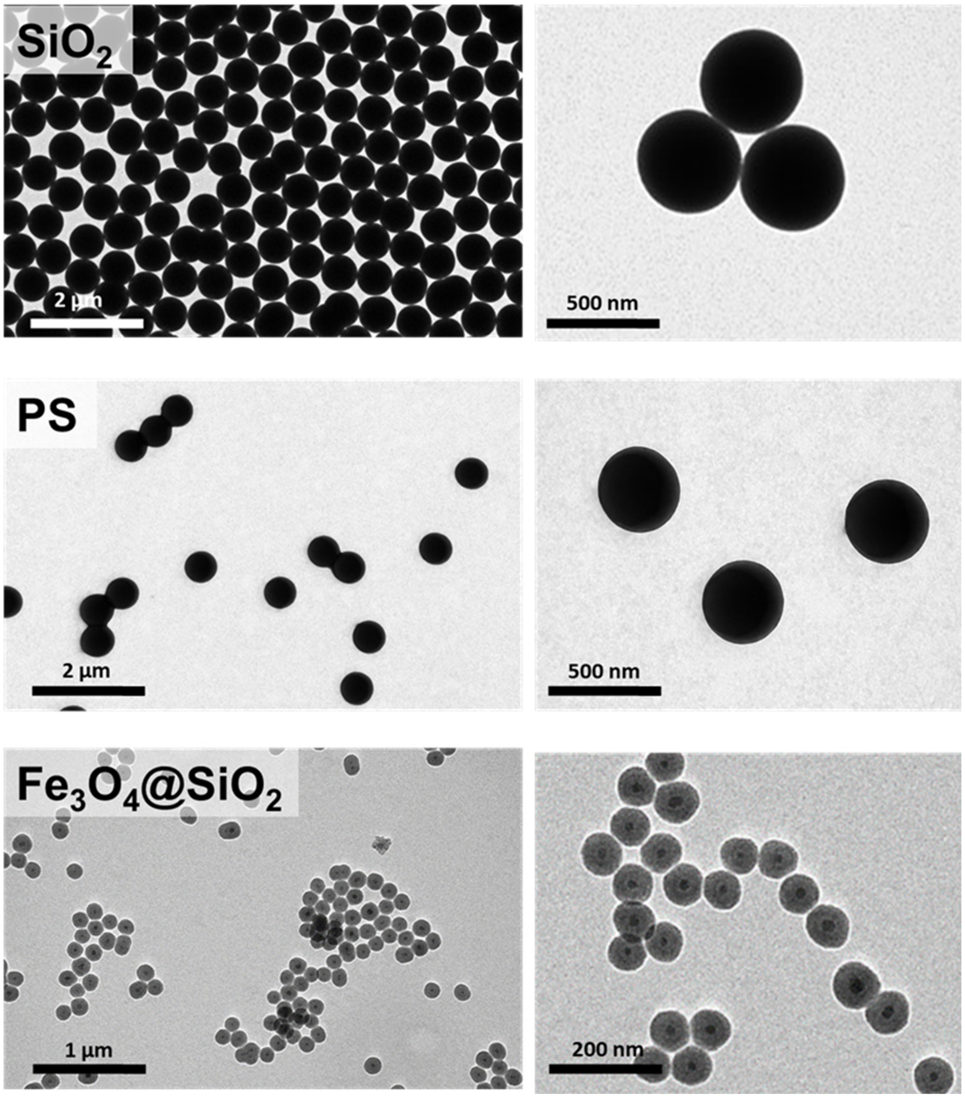

Figure S2. Transmission Electron Microscopy images of the nanoparticles used in the study. See Methods section of nanoparticle characterization.

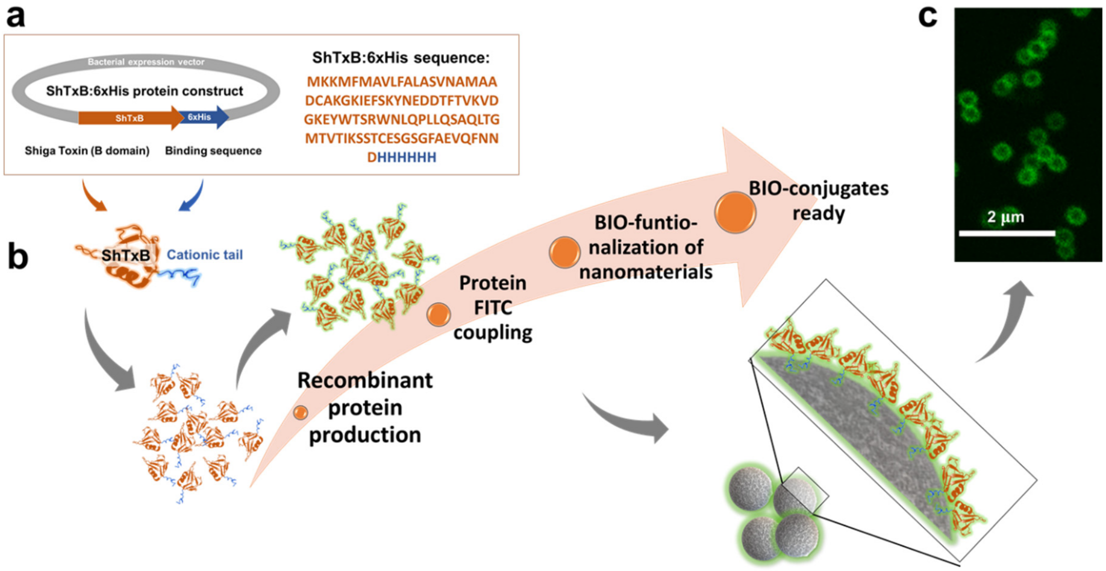

Figure S3. Ligand-protein production and nanomaterial functionalization procedure.

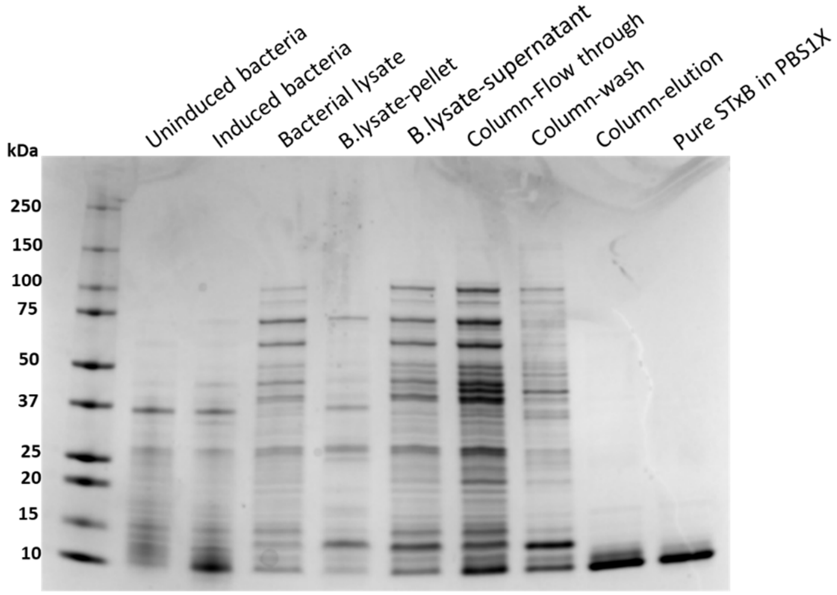

Figure S4. Coomassie-blue stained SDS-PAGE electrophoresis gel showing the different fractions of proteins obtained during the overexpression and purification steps of the StxB:6xHis protein (ShTxB).

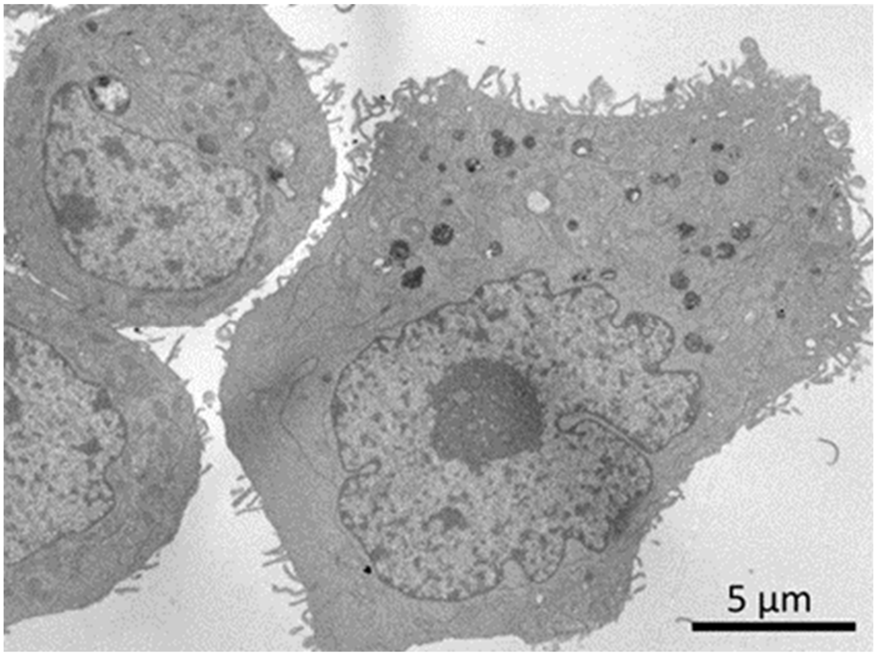

Figure S5. Transmission Electron Microscopy image of an ultrathin section of a control human HNC cell.
